# Primary irritant and delayed-contact hypersensitivity reactions to the freshwater cyanobacterium *Cylindrospermopsis raciborskii *and its associated toxin cylindrospermopsin

**DOI:** 10.1186/1471-5945-6-5

**Published:** 2006-03-31

**Authors:** Ian Stewart, Alan A Seawright, Philip J Schluter, Glen R Shaw

**Affiliations:** 1 National Research Centre for Environmental Toxicology, University of Queensland, 39 Kessels Road, Coopers Plains, QLD 4108, Australia; 2 School of Population Health, University of Queensland, Herston Road, Herston, QLD 4006, Australia; 3 Cooperative Research Centre for Water Quality and Treatment, PMB 3, Salisbury, SA 5108, Australia; 4 Faculty of Health and Environmental Sciences, Auckland University of Technology, Private Bag 92006, Auckland 1020, New Zealand; 5 School of Public Health, Griffith University, University Drive, Meadowbrook, QLD 4131, Australia

## Abstract

**Background:**

Freshwater cyanobacteria are common inhabitants of recreational waterbodies throughout the world; some cyanobacteria can dominate the phytoplankton and form blooms, many of which are toxic. Numerous reports in the literature describe pruritic skin rashes after recreational or occupational exposure to cyanobacteria, but there has been little research conducted on the cutaneous effects of cyanobacteria. Using the mouse ear swelling test (MEST), we sought to determine whether three toxin-producing cyanobacteria isolates and the purified cyanotoxin cylindrospermopsin produced delayed-contact hypersensitivity reactions.

**Methods:**

Between 8 and 10 female Balb/c mice in each experiment had test material applied to depilated abdominal skin during the induction phase and 10 or 11 control mice had vehicle only applied to abdominal skin. For challenge (day 10) and rechallenge (day 17), test material was applied to a randomly-allocated test ear; vehicle was applied to the other ear as a control. Ear thickness in anaesthetised mice was measured with a micrometer gauge at 24 and 48 hours after challenge and rechallenge. Ear swelling greater than 20% in one or more test mice is considered a positive response. Histopathology examination of ear tissues was conducted by independent examiners.

**Results:**

Purified cylindrospermopsin (2 of 9 test mice *vs*. 0 of 5 control mice; p = 0.51) and the cylindrospermopsin-producing cyanobacterium *C. raciborskii *(8 of 10 test mice *vs*. 0 of 10 control mice; p = 0.001) were both shown to produce hypersensitivity reactions. Irritant reactions were seen on abdominal skin at induction. Two other toxic cyanobacteria (*Microcystis aeruginosa *and *Anabaena circinalis*) did not generate any responses using this model. Histopathology examinations to determine positive and negative reactions in ear tissues showed excellent agreement beyond chance between both examiners (*κ *= 0.83).

**Conclusion:**

The irritant properties and cutaneous sensitising potential of cylindrospermopsin indicate that these toxicological endpoints should be considered by public health advisors and reservoir managers when setting guidelines for recreational exposure to cyanobacteria.

## Background

Cyanobacteria, also known as blue-green algae, are common inhabitants of freshwater lakes and reservoirs throughout the world. Under favourable conditions, certain cyanobacteria can dominate the phytoplankton within a waterbody and form nuisance blooms. Anecdotal and case reports have documented skin rashes associated with contact exposure to freshwater cyanobacteria, from recreational and occupational settings [1]. Some reports and health advisories refer to irritant reactions [[2,3] (p.32), [4]], but there are also convincing reports of hypersensitivity reactions [5,6]. By way of contrast, some cyanobacterial products appear to possess dermal anti-inflammatory properties [7,8]. We present here the first report of pathological effects on mammalian skin by the alkaloid cyanotoxin cylindrospermopsin.

There is a small body of literature relating to research into the cutaneous effects of planktonic freshwater cyanobacteria and the toxins of freshwater cyanobacteria. Human clinical studies are discussed in the accompanying paper by Stewart *et al *[9]. Only two reports referring to experimental cutaneous reactions to toxic freshwater cyanobacteria using animal models were found in the literature: van Hoof *et al *[10] reported no adverse effects in two experiments involving rabbits and guinea pigs. Torokne *et al *[11] suggest that exposure to heterotrophic bacterial lipopolysaccharides may explain some of their positive findings from non-axenic cynanobacterial preparations. Krishnakumari *et al *[12] reported dermal testing of rats with *Spirulina platensis*, a non-toxic cyanobacterium; no signs of erythema or oedema were observed. Shirai *et al *[13] describe an intraperitoneal induction and subcutaneous challenge test for delayed-type hypersensitivity in mice, examining responses to non-toxic *Microcystis *sp., with positive findings reported. Wannemacher *et al *[14] used guinea pigs to determine acute dermal toxicity of microcystin-LR (MC-LR) and saxitoxin, with dimethyl sulfoxide (DMSO) as a vehicle. Dermal LD_50_s were reportedly similar to or lower than rodent oral LD_50_s.

In their study of the cutaneous effects of axenic cyanobacteria, bloom samples and purified microcystin-LR, Torokne *et al *[11] conducted intradermal irritancy tests on rabbits; results were described as slight or negligible. Guinea pig maximisation tests were used for DCH determination; the reported proportion of sensitised animals ranged from 30% from two *Microcystis *blooms to 91% from an *Aphanizomenon *bloom. A non-axenic *C. raciborskii *preparation reportedly sensitised 50% of guinea pigs. These proportions were applied to allergen rating scales of "moderate allergen" for the *Microcystis *and *C. raciborskii *preparations to "extremely strong allergen" for the *Aphanizomenon *preparation [11].

Some difficulties in interpreting this work come from the reported percentages of sensitised guinea pigs, which are not derivable from the reported number of animals tested. Also, no details are given about the grading of end-points (erythema and oedema) in either test animals or controls; no photographic evidence of positive and negative reactions was published. The authors noted that no irritant or DCH effects were seen with axenic cyanobacterial preparations, and therefore concluded that the lipopolysaccharides (LPS) of contaminating bacteria were implicated in their positive findings from non-axenic cyanobacteria [11]. The attribution of LPS involvement – cyanobacterial or otherwise – in skin pathology is uncertain at best, and the only references to reports of LPS-associated acute skin rashes appear to be from the cyanobacteria literature, with no specific research findings to support such attribution. The topic of cyanobacterial lipopolysaccharides and their pathogenicity was recently reviewed by Stewart *et al *[15]. If the findings of Torokne *et al *[11] can be repeated in other laboratories, supporting evidence such as histopathological examination of challenged skin would be helpful.

Shirai *et al *[13] describe an intraperitoneal induction and subcutaneous challenge test for delayed-type hypersensitivity, examining responses to non-toxic *Microcystis *bloom samples and an axenic non-toxic culture of *M. aeruginosa*. Mice were challenged two weeks after i.p. inoculation of cells by s.c. injection into a rear footpad, and increases in footpad thickness were measured. The authors report induction of delayed-type hypersensitivity by this mouse footpad swelling test, although it is not immediately clear from the methods and reported results how the authors demarcated irritant reactions from DCH reactions. Control mice reportedly showed increased footpad swelling, although the report does not state which material was administered to controls. We also have some concerns regarding the method, as there is no description of how footpad thickness was measured, and there was apparently no injection of solvent into the contralateral footpad, so it is not known whether or to what degree the challenge injection procedure may have contributed to footpad swelling [13].

Wannemacher *et al *[14] used guinea pigs to determine acute dermal toxicity of microcystin-LR (MC-LR) and saxitoxin, as well as other eukaryotic algal toxins and T2 mycotoxin. Toxins were dissolved in dimethyl sulfoxide (DMSO) and applied to shaved dorsal skin. The dermal LD_50_s for saxitoxin and MC-LR were reported at 300 μg/kg and 2 mg/kg respectively. These results are surprising, given that oral LD_50_s for MC-LR are reported as ranging from 3 mg/kg to 11 mg/kg in mice, and higher in rats [16,17]. Oral LD_50_s for saxitoxin range from 64 μg/kg in neonatal rats, to 530 μg/kg in adult rats, and 260 μg/kg in mice [16]. The use of DMSO as a vehicle may have contributed significantly to the trans-dermal transport of these highly water-soluble toxins into the circulation, hence the results of these experiments may not be readily interpretable from a human health risk perspective.

Van Hoof *et al *[10] and Krishnakumari *et al *[12] appear to have confused the concepts of irritancy and allergenicity in their reports. Both studies apparently were not designed to incorporate separate induction and challenge phases to elicit delayed-contact hypersensitivity, i.e. they were short-term tests for irritancy, yet the authors report their findings (which were negative in each case) in terms of DCH [10] and cutaneous allergy [12].

The MEST was chosen for these studies as a model to investigate sonicated suspensions of lyophilised freshwater cyanobacteria, with the reported advantages over guinea-pig models of cost-effectiveness, reduced test duration and objective data generation as opposed to visual scaling [18]. Briefly, the procedure involves four applications of test material to the abdomen – the induction phase – followed by challenge application to the ear. A micrometer gauge is used to measure ear thickness; an increase in ear swelling of 20 per cent or greater in one or more test mice is regarded as a positive result [19]. Using the MEST, we sought to determine whether three toxin-producing cyanobacteria isolates and the purified cyanotoxin cylindrospermopsin were capable of generating delayed-contact hypersensitivity reactions.

## Methods

Three sonicated cyanobacterial suspensions were tested by separate MEST procedures:

• *Microcystis aeruginosa *- strain QH/NR/Ma/03/3 from the Queensland Health Scientific Services Culture Collection. This strain produces microcystins, predominantly microcystin-LR (MC-LR). Microcystins are a group of >60 structurally related cyclic peptide toxins; microcystins in this suspension were measured at 13.6 mg/L total microcystins expressed as MC-LR.

• *Anabaena circinalis *- strain AWQC Gar 311FR from the Australian Water Quality Centre, Adelaide. This strain produces saxitoxins, which are structurally related alkaloid neurotoxins, measured at 6.0 mg/L total saxitoxins in this suspension.

• *Cylindrospermopsis raciborskii *strain AWT 205 isolated and cultured from a Sydney ornamental lake [20] and supplied by Dr Peter Hawkins of Australian Water Technologies, Sydney. This strain produces the alkaloid toxin cylindrospermopsin, measured at 73 mg/L in this suspension.

Growth, harvesting, lyophilisation and storage of cells were conducted as described in the accompanying paper by Stewart *et al *[9]. *A. circinalis *was grown and harvested as per *M. aeruginosa *cells in 20 L batch culture.

Cyanobacteria preparations were made by mixing 200 mg lyophilised cells in 10 mL 70%v/v ethanol in Milli-Q^® ^filtered water to produce 2%w/v suspensions. Cell suspensions were steeped overnight at 4°C. Cell integrity was disrupted by subjecting each suspension to ultrasonic pulsing for 30 seconds, using a Branson Ultrasonics Sonifier 450 instrument.

Saxitoxins were analysed by high performance liquid chromatography (HPLC) with fluorescence detection using a Shimadzu LC-10AVP system based on the methods of Lawrence *et al *[21]; microcystins were measured using a Shimadzu LC-10A HPLC with photodiode array detection using the methods of Lawton *et al *[22]. Cylindrospermopsin was quantified by HPLC-MS/MS with a Perkin Elmer series 200 HPLC coupled to a PE SCIEX API 300 mass spectrometer [23].

Cylindrospermopsin was produced from strain AWT 205, grown in continuous culture as described in Stewart *et al *[9], isolated by solid phase extraction, purified by preparative HPLC and quantified by HPLC-MS/MS as described above. Both 100 μg/mL and 50 μg/mL solutions in 70%v/v ethanol/water were prepared.

A 0.1%w/v dinitrochlorobenzene (1-chloro-2,4-dinitrobenzene, purchased from Sigma-Aldrich P/L) in 70%v/v ethanol/water solution was used for a positive control MEST. All suspensions and solutions were stored at -20°C prior to use.

Specific-pathogen-free female Balb/c mice were supplied by either Laboratory Animal Services, University of Adelaide, or bred in-house at QHSS Biological Research Facility. Mice were between 8 and 10 weeks of age, housed in groups of five with free access to food and water. Standard rodent chow enriched with vitamin A acetate 250 IU/g (Glen Forest Stock Feeds, Western Australia) was supplied for two weeks prior to testing and throughout the duration of each experiment. Following removal of abdominal fur all mice received bilateral intradermal injections of Freund's complete adjuvant (Sigma-Aldrich P/L). Abdominal and ear skin was dried with a domestic mains-powered hair dryer after application of test materials and vehicle controls. Ear thickness was measured with a micrometer gauge (Model 2046 F, Mitutoyo Corp., Tokyo).

The MEST experiments for DCH were conduced according to the methods of Gad [19], with the following modifications:

1. The test ear of each mouse was randomly assigned as either the left or right ear. This simple modification should increase the reliability of the model by allowing the investigator to be blinded to the identity of test ears, provided that test materials do not affect the colour of test solutions.

2. Individual mice were identified by tail markings using a permanent marker pen. This is necessary for research purposes in order to allow random allocation of test ears, and for histopathological examination of ears.

3. Abdominal fur was removed with a proprietary depilatory cream (Nair brand. Active ingredient: calcium thioglycolate <10%w/w). Tape stripping to remove the stratum corneum was performed on days 1, 3 and 5 (i.e. tape-stripping was not conducted on day 0, after depilatory treatment).

4. Mice were anaesthetised for ear thickness measurements with a 50:50 mixture of CO_2 _and O_2_, as described by Blackshaw & Allan [[24] (p.73)].

5. Pre-test screens for irritancy were not conducted in order to reduce the number of animals used. Scoping experiments on mice conducted in our laboratory to investigate irritancy and to familiarise ourselves with the MEST techniques led us to suspect that toxic *M. aeruginosa *and non-toxic *A. circinalis *would not initiate irritant responses when administered at high concentrations (data not shown).

At least two, and more frequently three micrometer readings were taken on each ear, depending on the time that each mouse remained motionless after CO_2_/O_2 _anaesthesia. The average of the two or three readings taken on each ear was used to determine differences in ear thickness between test ears and control ears. Percent ear swelling at each measurement period was determined by the formula: % ear swelling = 100*(A-B)/B where A denotes the mean test ear thickness and B denotes the mean control ear thickness.

All challenge and rechallenge concentrations were the same as for the induction doses (2%w/v lyophilised cyanobacteria in 70%v/v ethanol), with the exception of the cylindrospermopsin MEST. In this test, the challenge dose was 20 μL to the ventral pinna and 20 μL to the dorsal pinna of each test ear using a 50 μg/mL CYN in 70% ethanol solution, i.e. half the concentration of the induction dose. CYN rechallenge doses were performed with the normal induction concentration of 100 μg/mL CYN in 70% ethanol.

### Histopathology

Mice were sacrificed by CO_2 _inhalation; ears from five test mice and five control mice in the CYN MEST were removed and fixed in 10% phosphate-buffered formalin. Ears from test mice that produced positive MEST responses were selected for histopathology examination. Ears from test mice in the *C. raciborskii *MEST were not considered in this examination as these mice were subjected to ear thickness measurements beyond day 19, i.e. past the 48-hour post-rechallenge period, in order to monitor their return to baseline. However, ears from three *C. raciborskii *MEST control mice were collected after the 48-hour post-challenge measurements and prepared on microscopy slides. Ears from two mice in the *A. circinalis *and *M. aeruginosa *MESTs were also collected. Liver, kidney, spleen and lung tissue were harvested from three mice that were peak responders in each of the *C. raciborskii *and CYN MESTs; these organs were fixed as for ears. Tissue slices were stained with haematoxylin and eosin and mounted on glass slides for microscopic examination.

Two examiners conducted histopathological examinations: 1^st ^examiner was author AAS; 2^nd ^examiners were an expert pathologist with specific expertise in pathology of the skin, plus a consultant dermatologist and three dermatology registrars. 2^nd ^examiners reported each slide by consensus. Histopathological examination was conducted by presenting each pathologist with identified slides from one control ear and two test ears from the positive control MEST (0.1% DNCB) to allow for familiarisation with normal mouse ear morphology and type-IV hypersensitivity-related changes. All subsequent slides were read with the examiners blinded to their identity.

### Statistical analyses

Fisher's exact test was used to compare proportions of test ear increases greater than 20% between test and control mice. Histopathology inter-rater agreement was assessed using Cohen's kappa (*K*) statistic. Fisher's exact test was used to examine the marginal homogeneity of the pathologist responses by comparing the unmatched distribution of equivocal / normal / positive ratings. Statistical tests were performed with SPSS v.13.0, and p < 0.05 was used to denote statistical significance.

### Ethical approval

QHSS Animal Ethics Committee granted approvals for the original study protocol and all subsequent amendments under clearance number NRC 3/98/20 IS.

## Results

### MESTs for delayed-contact hypersensitivity

**Figure 1:**
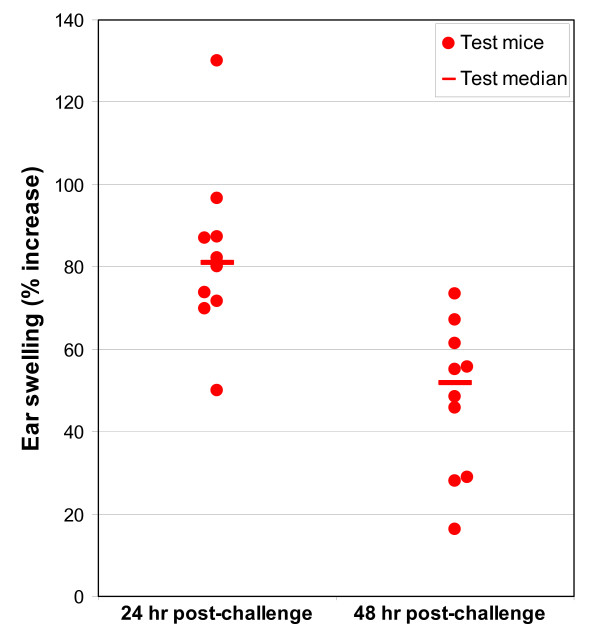
Point plot showing individual mouse ear thickness changes: DNCB MEST (positive control experiment).

**Figure 2:**
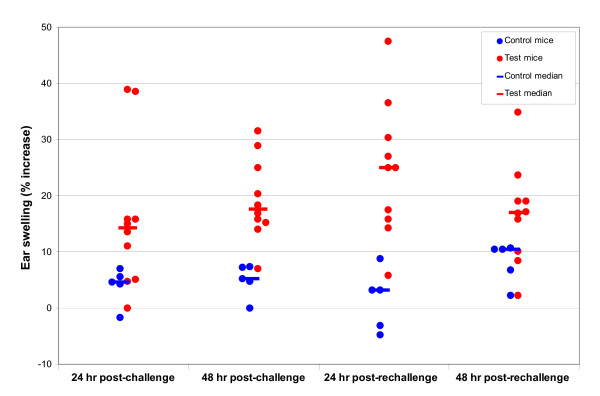
Point plot showing individual mouse ear thickness changes: *C. raciborskii *MEST (suspension contained 73 μg/mL CYN).

**Figure 3:**
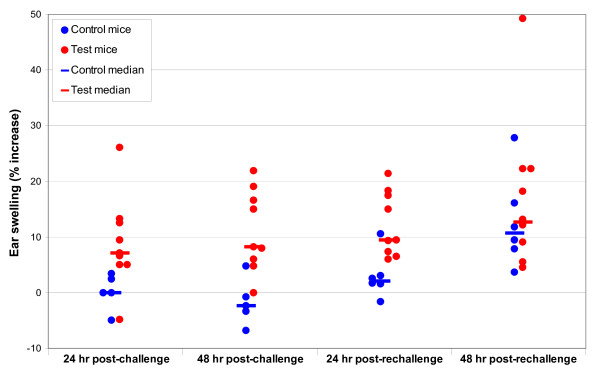
Point plot showing individual mouse ear thickness changes: cylindrospermopsin MEST.

Ear thickness increases, expressed as percent ear swelling, for the four cyanobacterial MESTs (*M. aeruginosa*, *C. raciborskii*, *A. circinalis *and purified cylindrospermopsin) and the positive control MEST (0.1% DNCB) are given in Table 1. Figures 1, 2, 3 are point plots showing ear thickness changes of individual mice in MESTs for DNCB, *C. raciborskii *and CYN. All mice dosed with DNCB registered ear thickness increases greater than 20%. Test ears were erythematous; some had obvious oedema. No mice dosed with 2%w/v suspensions of *A. circinalis *or *M. aeruginosa *reached 20% ear swelling. The MEST using a 2%w/v suspension of *C. raciborskii *containing 73 μg/mL cylindrospermopsin (CYN) resulted in a clear median increase in ear swelling of test animals; eight of ten test mice produced ear thickness increases of greater than 20% at challenge and/or rechallenge; no control mice gave ear thickness increases of >20%. The MEST for purified CYN produced ear thickness increases greater than 20% in two of nine test mice and none of five control mice following challenge doses of 50 μg/mL CYN. After rechallenge with 100 μg/mL CYN, four test mice and one of six control mice had ear thickness readings of >20%.

### Induction-phase reactions – *C. raciborskii *suspension and CYN

**Figure 4:**
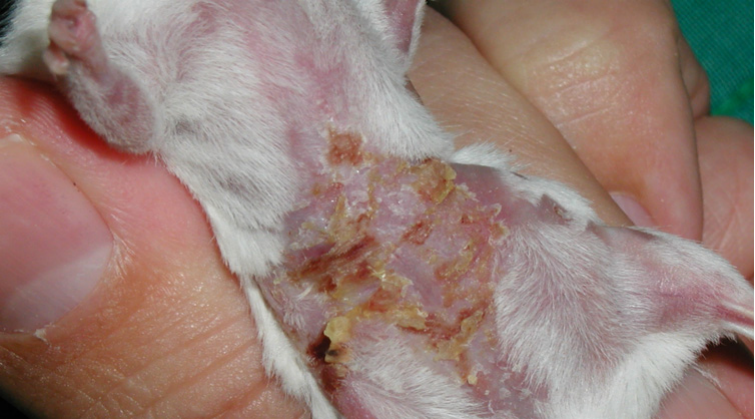
Reaction seen at induction phase – cylindrospermopsin 100 μg/mL in 70% ethanol.

**Figure 5:**
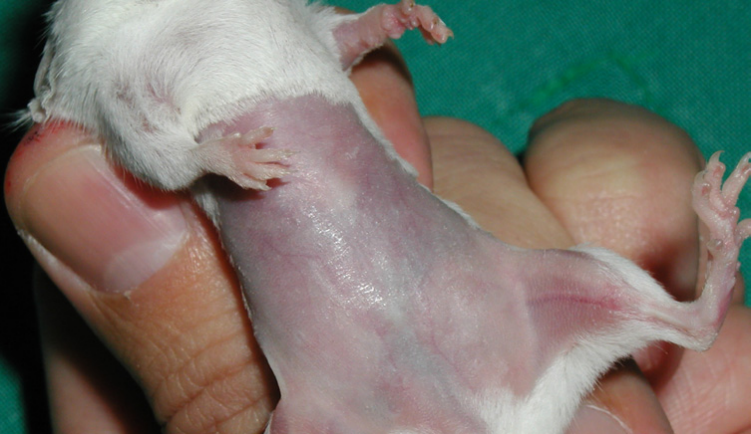
Cylindrospermopsin MEST control mouse at induction phase – dosed with vehicle (70% ethanol)

As well as the ear thickness changes given in Table 1 and Figures 2 and 3, primary irritant reactions were seen on the abdominal skin of mice tested with the *C. raciborskii *suspension, and with purified CYN. No signs of irritancy were seen on the abdominal skin of mice treated with suspensions of *M. aeruginosa *or *A. circinalis*, or DNCB solution. The reaction seen on *C. raciborskii *and CYN-treated mice occurred on all test mice with varying degrees of severity. Figure 4 shows a test mouse with this reaction. Figure 5 shows a control mouse for comparison; vehicle only (70%v/v ethanol/water) was applied to the abdominal skin of control mice.

Lesions produced on abdominal skin were noted from the second induction day onwards. Abdominal skin was dry, with yellowish/brown crusts and desquamation seen especially in inguinal areas and on upper hind limbs. Only very gentle tape-stripping was required in order to produce shiny abdominal skin, and in some mice with these lesions, tape stripping was not performed when evidently painful (manifested by shrill vocalising and struggling). These lesions did not appear to be chronically painful when not disturbed, as affected mice were observed to be eating, sleeping and using their exercise wheel. More severely affected mice walked with an unusual gait, holding their abdomens clear from cage bedding. This was presumably in order to avoid contact by wood shavings with encrusted lesions, which, when dislodged, resulted in blood or serous fluid oozing from exposed skin. Healing was very rapid after induction dosing was complete; by the completion of the experiments at re-challenge (day 17), abdominal skin appeared essentially normal, with minor residual scarring seen on two animals.

### Histopathology

#### 1^st ^examiner

Test ears from two mice dosed with 0.1% DNCB were described as presenting with hyperaemia, infiltration of inflammatory cells – mainly mononuclear cells with occasional polymorphonuclear leukocytes (PMNs); dilated lymphatic vessels and oedema (increased space between connective tissue). Typical findings from the test ears of test mice dosed with cylindrospermopsin were: oedema, thickening, and inflammatory cell infiltration – mainly mononuclear cells, though varying degrees of PMN infiltration were also seen. One test ear slide showed an epidermal lesion, with hyperkeratinisation and associated PMN infiltration. The overall impression was of mostly mild inflammatory reactions to cylindrospermopsin, with occasionally more severe effects. No pathological changes were seen in any of the organs (liver, spleen, kidney, lung) from mice cutaneously exposed to *C. raciborskii *suspension or cylindrospermopsin.

#### 2^nd ^examiners

Test ears from the two mice dosed with DNCB were described as inflamed, and rated "+++". One ear from a cylindrospermopsin-challenged mouse was rated "+++ with eosinophilia", and a control ear (i.e. vehicle-only treated) from another cylindrospermopsin-challenged mouse was rated "++ with some eosinophils". Again, liver, kidney, lung and spleen tissues from all six mice dosed with *C. raciborskii *suspension or purified cylindrospermopsin were reported as normal.

#### Measures of inter-rater agreement

Table 2 presents results of microscopy examinations categorised into positive, negative and equivocal findings. There was no difference in the marginal distributions of the categories between raters (p = 0.35) and there was excellent agreement beyond chance (*κ *= 0.83, 95% confidence interval: 0.61, 1.0).

**Figure 6:**
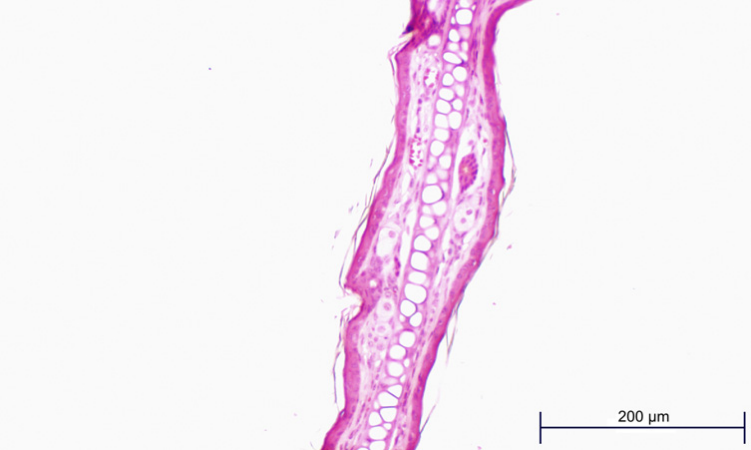
Cylindrospermopsin MEST, control ear (vehicle only).

**Figure 7:**
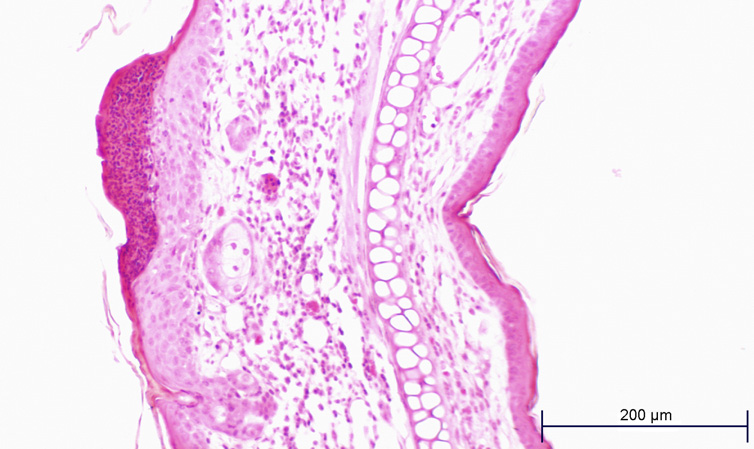
Cylindrospermopsin MEST, test ear.

Figures 6 and 7 are examples of histopathology findings. Both slides (test ear and control ear) were prepared from ears taken from the same mouse. Note the test ear inflammatory cell infiltrate, thickening of dermal structures and the area of epithelial hyperplasia.

## Discussion

Before commencing these studies we conducted modified MEST experiments to investigate primary irritant reactions, as described by Patrick *et al *[25] and Patrick *et al *[26]. We examined responses to suspensions of *M. aeruginosa *(QH/NR/Ma/03, 5%w/v lyophilised cells in 75% methanol) and *A. circinalis *(non-toxic bloom sample, Gordonbrook Dam, Queensland; 10% w/v in 75% methanol). These were highly concentrated cyanobacterial preparations, being about the maximum amounts of lyophilised cyanobacteria that could produce viscous suspensions. No positive findings were observed or measured (data not presented).

Both MESTs for DCH using toxic *M. aeruginosa *and *A. circinalis *suspensions were unremarkable in all respects; no abnormality was seen on either abdominal or ear skin, and no ear thickness measurements were greater than the threshold 20% increase. Therefore we did not proceed with any further investigations into the cutaneous effects of the cyanotoxins produced by these cyanobacterial isolates (MC-LR and saxitoxins respectively).

The MEST to investigate *C. raciborskii *was, by contrast, a very different experimental outcome. It became clear on day three of induction dosing, i.e. some 72 hours after the first induction dose, that a positive reaction was occurring. Dry, encrusted lesions were seen on the abdomens of all test animals – and on no control animals – from day three onwards. No abnormalities were seen on day one, i.e. 24 hours after the first dose. No observations were taken on day two, following the second induction dose, so it is not known whether these abdominal lesions would have been apparent any earlier than when first observed at 72 hours, following two dosings at 0 and 24 hours. Aware of the possibility that the reactions seen on abdominal skin would also be found on the test ears of control (i.e. naïve to *C. raciborskii*) mice, thus invalidating the MEST, we decided to proceed with the test. No such reaction was seen (see Table 1) in any of the ten control mice used in challenge and rechallenge tests, thus confirming the findings of Gad [19], who notes that a concentration of test material that is irritating to one site – abdomen or ear – may not be an irritant concentration for the other. Irritant concentrations for ears are frequently higher than concentrations that will irritate abdominal skin [18]. Therefore this MEST has shown that a 2%w/v suspension of cylindrospermopsin-producing *C. raciborskii *produces both a primary irritant reaction on mouse abdominal skin, and delayed-contact hypersensitivity. Unilateral erythema and swelling was seen on the ears of some test mice, similar to but of lesser intensity than the reactions seen on positive control MEST mice dosed with 0.1% DNCB. No such reactions were seen on any control mouse ears. No signs of cutaneous injury similar to that seen on abdominal skin were observed on any ears. Reactions were more pronounced after rechallenge compared to challenge readings, as shown in Table 1 and Figure 2.

Another MEST experiment using purified cylindrospermopsin was conducted, to investigate further the findings from the *C. raciborskii *MEST. The concentration was higher than the measured concentration of cylindrospermopsin in the 2% *C. raciborskii *suspension (100 μg/mL *cf*. 73 μg/mL), so when irritant reactions on the abdominal skin of test mice were seen during the induction phase, the possibility that irritant reactions might be induced on the test ears of control mice, thus invalidating the experiment, again needed to be considered. Therefore the challenge concentration of cylindrospermopsin was reduced to 50 μg/mL (i.e. half that of the induction dose). Rechallenge was conducted with the induction concentration of 100 μg/mL, but one of six control mice produced an ear swelling of 28%, therefore no clear statement can be made about these MEST rechallenge readings, as 100 μg/mL cylindrospermopsin is most likely an irritant dose for the mouse ear. The effect size for the CYN MEST (control mice: p1 = 0/5 = 0; test mice: p2 = 2/9 = 0.22) indicates that the statistical power to determine a significant difference of this size is less than 5%.

Reactions seen on abdominal skin in the cylindrospermopsin MEST were identical to those seen during the *C. raciborskii *MEST. Again, all test mice had visible lesions to varying degrees. Healing was rapid after the final induction dose on day 5, so that by day 8, one mouse had a residual dry scab on the abdomen, and the remaining eight mice had essentially normal looking abdominal skin. Because of the different challenge dose used in the cylindrospermopsin MEST and the probable irritant reactions seen at rechallenge, it is not possible to make confident predictions about the relative contribution of cylindrospermopsin to the reactions seen in the *C. raciborskii *MEST.

The number of mice used in the CYN MEST was too small to detect a statistically significant difference between proportions of test and control mice achieving the threshold ear thickness increases after challenge alone. However, the gross similarity of reactions seen on abdominal skin in both tests, and the fact that two test mice in the CYN MEST challenge test met the methodological criteria of Gad [19] for delayed-contact hypersensitivity (i.e. a statistically non-significant but biologically significant finding) suggests that cylindrospermopsin is the major contributor to the outcome of the *C. raciborskii *MEST.

There are several specific points of departure when attempting to apply the results of these findings to assessing the risk for humans in recreational or occupational contact with cylindrospermopsin-producing cyanobacteria. These being the concentration of cylindrospermopsin was well above that likely to be encountered in natural waters (see following discussion); barrier function was disrupted by tape-stripping during the induction phase (removal of the stratum corneum by tape-stripping stimulates the release of pre- formed IL-1α and other inflammatory cytokines from the epidermis [27,28]); a vehicle (70% ethanol) was used to aid the transport of soluble factors across the epidermis (matrix effects can be highly significant determinants of the potency of allergens, with reported variability of more than two orders of magnitude due to different vehicles [29]); and mice were immunologically primed to respond to sensitisers by use of Freund's adjuvant and hypervitaminosis-A (vitamin A induces epidermal hyperplasia in mice; most cell-mediated immune responses are stimulated by vitamin-A supplementation [30]). However, the main aim of conducting animal models in this kind of exploratory work was to determine whether the mechanism of irritancy and hypersensitivity was feasible with these cyanobacterial preparations. Further investigation to determine dose-response relationships and cellular and molecular mechanisms should follow from this work.

### Histopathology

Histopathology findings apparently revealed three false positive readings: firstly, the test ears from two control mice tested with 100 μg/mL purified cylindrospermopsin at rechallenge, i.e. test material applied to naïve mice. However, from Table 1 and Figure 3 it seems clear that the rechallenge CYN concentration of 100 μg/mL was an irritant dose for at least one mouse, as determined by an ear thickness increase greater than 20 per cent. Both pathologists rated the same two test ears as positive from this group of five control mice. One of these "false positive" test ears was from the mouse that produced a 28% difference in ear thickness after rechallenge (Table 1 & Figure 3). Therefore, because both examiners rated the same two ears as having a positive reaction, and the dose applied to the ears of these naïve mice was probably in the irritant range, the most likely interpretation is that these were not false positive readings but true positive findings. The 1^st ^examiner described the slide from the mouse producing the 28% ear swelling reading as having "oedema, mild inflammatory infiltrate, with some polymorphs", and the 2^nd ^examiner group rated this ear at "+++".

The other "false positive" reading is from a control ear, i.e. dosed with vehicle only. The 1^st ^examiner was twice presented with this slide; he reported it positive on both occasions, describing it as oedematous, with polymorph infiltration. The 2^nd ^examiner group also described this slide as positive, rating it "++ with some eosinophils". Therefore, it would appear that this slide also represented true positive histopathology. Ear thickness measurements on this mouse throughout the MEST procedure were always positive, i.e. the test ear consistently produced higher micrometer readings than the control ear in question. Care was taken during the MEST procedure to prevent incorrect dosing of test and control ears: the test ears of all mice in each MEST were dosed and dried, then the container with the test suspension or solution was sealed, the container tube for the vehicle was opened, and each mouse was taken from its cage for dosing of control ears. The histopathology of the test ear from this mouse was also clearly positive: the 1^st ^examiner described it as having cellular proliferation, polymorphs and mononuclear cells and oedema; the 2^nd ^examiner group rated this test ear at "+++".

No positive histopathology was seen in test ears of mice from the *A. circinalis *and *M. aeruginosa *MESTs; this supports the negative findings of both those MESTs. In conclusion, the histopathology findings correlate well with the MEST results, and the two independent pathologist examinations demonstrate excellent agreement.

### Relative sensitising potency of cylindrospermopsin

Understanding the relative potency of a toxin is an important step in defining the associated public health risk. This also applies to safety assessments of skin sensitisers, which may vary up to 10,000-fold in sensitising potency [31]. The induction and sensitisation concentrations of CYN in the *C. raciborskii *and CYN MESTs (50, 73 and 100 μg/mL) were artificially high, with field concentrations in lakes and ponds typically less than 1 mg/L [32-34]. However, when considering these concentrations in the context of thresholds for sensitising chemicals proposed by the European Centre for Ecotoxicology and Toxicology of Chemicals (ECETOC) [31], 50 μg/mL (0.005%w/v) and 100 μg/mL (0.01%w/v) are in the range of concentrations to be classified as "extreme" sensitisers. Therefore, DCH generated by CYN at any concentration below those used in these MESTs will, by the ECETOC rating [31], still place cylindrospermopsin in the category of an extremely potent sensitiser.

Threshold concentrations can be determined for chemical sensitisers, and dose-response relationships are apparent in both induction and sensitisation phases [[29,31,35] (pp.2, 3, 17)]. If dose-response studies show that cylindrospermopsin is capable of eliciting DCH at lower concentrations than the artificially high doses used in these experiments, the appropriate question will be whether CYN at naturally occurring levels is below the threshold for DCH – and irritant reactions.

## Summary and conclusion

Using a mouse model of delayed-contact hypersensitivity, we have demonstrated that both a cylindrospermopsin-containing suspension of *C. raciborskii *and purified cylindrospermopsin elicit irritant and hypersensitivity reactions. Toxin-producing suspensions of *M. aeruginosa *and *A. circinalis *failed to induce any observable or measurable adverse reactions. All three sonicated preparations were applied to mouse skin at identical concentrations (2%w/v lyophilised cyanobacteria). Further experimental work should follow in order to confirm positive and negative findings of this study and previously published work. Studies of the cellular and molecular processes involved in the cutaneous toxicity of cylindrospermopsin would be of great interest.

Cylindrospermopsin-producing cyanobacteria are widely distributed in tropical, subtropical and temperate waters of all continents. Public health authorities and recreational water managers are aware of the potential for harm through accidental or deliberate ingestion of water contaminated by cylindrospermopsin; authorities may also need to consider the potential for cylindrospermopsin-producing cyanobacteria to be associated with complaints of acute skin reactions.

## Abbreviations

CYN cylindrospermopsin

DCH delayed-contact hypersensitivity

DMSO dimethyl sulfoxide

DNCB dinitrochlorobenzene

ECETOC European Centre for Ecotoxicology and Toxicology of Chemicals

HPLC high performance liquid chromatography

HPLC-MS/MS HPLC + tandem mass spectrometry

i.p. intraperitoneal

LD_50 _lethal dose for 50% of test animals

LPS lipopolysaccharide(s)

MC-LR microcystin-LR

MEST mouse ear swelling test

PMN polymorphonuclear leukocyte

QHSS Queensland Health Scientific Services

s.c. subcutaneous

## Competing interests

The author(s) declare that they have no competing interests.

## Authors' contributions

IS initiated the study conception and design, grew and processed cyanobacteria, conducted the animal experiments and statistical tests, and drafted the manuscript. AAS and GRS developed the initial irritancy study concept. AAS conducted histopathology examinations. PJS provided statistical advice. GRS and PJS supervised the project. All authors read and endorsed the final manuscript.

## Pre-publication history

The pre-publication history for this paper can be accessed here:



## Figures and Tables

**Table 1:** Mouse ear swelling tests (MESTs): positive control + three cyanobacteria suspensions + purified cylindrospermopsin

**TEST MATERIAL**	**Proportion of test mice with ear swelling >20% after challenge**	**Proportion of test mice with ear swelling >20% after re-challenge**	**Per cent ear swelling of test group after challenge. Median (range)**	**Per cent ear swelling of test group after re-challenge. Median (range)**	**Per cent ear swelling of highest responder control mouse after challenge and re-challenge**	**Fisher's exact test comparing proportion of ear thickness increases <20% between test and control mice**
**0.1% DNCB**	100% *n*= 10 at 24 hrs *n *= 9 at 24 & 48 hrs	Not done	68.6 (16.4 – 130.0)	Not done	Not done	
**2% w/v *M. aeruginosa *microcystins 13.6 μg/mL**	0	0	1.9 (-11.6 – 10.7)	1.2 (-1.6 – 10.0)	8.9	
**2% w/v *A. circinalis *saxitoxins 6.0 μg/mL**	0	0	0.0 (-6.8 – 8.2)	1.5 (-2.9 – 9.5)	11.7	
**2% w/v *C. raciborskii *cylindrospermopsin 73 μg/mL**	60% *n*= 2 at 24 hrs *n*= 4 at 48 hrs	60% *n *= 6 at 24 hrs *n *= 2 at 24 & 48 hrs	15.8 (0.0 – 38.9)	18.3 (2.2 – 47.5)	10.7	p = 0.001
100 μg/mL cylindrospermopsin*	22% *n *= 1 at 24 hrs *n *= 1 at 48 hrs	44% *n *= 1 at 24 hrs *n *= 3 at 48 hrs	8.1 (-4.8 – 26.1)	12.6 (4.5 – 49.2)	4.8 (challenge) 27.8 (re-challenge)	p = 0.51 (challenge) p = 0.58 (rechallenge)

* challenge concentration applied to test ears was 50 μg/mL. Rechallenge concentration was 100 μg/mL (= induction concentration).

**Table 2:** Histopathology results – mouse ear swelling test

	1^st ^examiner (author AAS)				
		Positive	Normal	Uncertain	Total
	Positive	7	0	0	7
**2**^nd^** examiners(DW & IR)**	Normal	2	21	2	25
	Uncertain	0	0	0	0
	Total	9	21	2	32
